# VMP1 Constitutive Expression in Mice Dampens Pancreatic and Systemic Histopathological Damage in an Experimental Model of Severe Acute Pancreatitis

**DOI:** 10.3390/ijms26073196

**Published:** 2025-03-29

**Authors:** Veronica Boggio, Claudio Daniel Gonzalez, Elsa Zotta, Alejandro Ropolo, Maria Ines Vaccaro

**Affiliations:** 1 Instituto de Bioquímica y Biología Molecular Prof. Alberto Boveris, Consejo Nacional de Investigaciones Científicas y Técnicas (CONICET), Universidad de Buenos Aires, Buenos Aires 1113, Argentina; boggiovero@gmail.com (V.B.); ezotta@gmail.com (E.Z.);; 2 Centro de Educación Medica e Investigaciones Clínicas (CEMIC), Hospital Universitario Saavedra, Buenos Aires 1431, Argentina; claudiodanielg@gmail.com

**Keywords:** selective autophagy, necrosis, transgenic mice, pancreas, lung, liver, kidney

## Abstract

Acute pancreatitis (AP) an inflammatory condition caused by the premature activation of pancreatic proteases, leads to organ damage, systemic inflammation, and multi-organ failure. Severe acute pancreatitis (SAP) has high morbidity and mortality, affecting the liver, kidneys, and lungs. Autophagy maintains pancreatic homeostasis, with VMP1-mediated selective autophagy (zymophagy) preventing intracellular zymogen activation and acinar cell death. This study examines the protective role of VMP1 (Vacuole Membrane Protein 1)-induced autophagy using ElaI-VMP1 transgenic mice in a necrohemorrhagic SAP model (Hartwig’s model). ElaI-VMP1 mice show significantly reduced pancreatic injury, including lower necrosis, edema, and inflammation, compared to wild-type (WT) mice. Biochemical markers (lactate dehydrogenase-LDH-, amylase, and lipase) and histopathology confirm that VMP1 expression mitigates pancreatic damage. Increased zymophagy negatively correlates with acinar necrosis, reinforcing its protective role. Beyond the pancreas, ElaI-VMP1 mice exhibit preserved liver, kidney, and lung histology, indicating reduced systemic organ damage. The liver maintains normal architecture, kidneys show minimal tubular necrosis, and lung inflammation features are reduced compared to WT mice. Our results confirm that zymophagy functions as a protective pathophysiological mechanism against pancreatic and extrapancreatic tissue injury in SAP. Further studies on the mechanism of VMP1-mediated selective autophagy in AP are necessary to determine its relevance and possible modulation to prevent the severity of AP.

## 1. Introduction

AP is a sudden and painful disease characterized by the premature activation of pancreatic secretory proteins, leading to inflammation and self-damage to the organ [1,2]. The revised Atlanta criteria classify AP based on type (interstitial edematous or necrotizing), severity (mild, moderately severe, or severe), and phase (early or late) [3]. Once activated, inflammation leads to vascular damage, endothelial dysfunction, and recruitment of leukocytes to the pancreas, potentially progressing to SAP, where mortality is high due to multi-organ failure, significantly impacting the quality of life [4]. The liver, kidneys, and lungs are particularly affected in severe cases, and their failure strongly predicts mortality [5].

Under physiological conditions, cholecystokinin (CCK) acts as a pancreatic secretagogue in acinar cells to induce pancreatic secretion. However, excessive stimulation of CCK receptors (CCK-R) disrupts vesicular transport, triggering intracellular activation of proteolytic enzymes and ultimately leading to the cell death characteristic of acute pancreatitis [6]. Hartwig et al. [7,8] described a rat model of necrohemorrhagic acute pancreatitis induced by CCK-R hyperstimulation with caerulein (CAE) and the trypsinogen activator enzyme enterokinase (EK). This model exhibits severe local and systemic organ injury with marked intrapancreatic protease activation and is considered a model of SAP. Protease activation plays a crucial role in triggering local and systemic inflammatory responses.

Autophagy, an early cellular event in AP that occurs after pancreatic protease activation [9], is an evolutionarily conserved process that degrades cytoplasmic components. Functioning as a nonselective bulk degradation mechanism during starvation promotes cell survival [10,11]. Beyond this, autophagy can selectively eliminate misfolded or protein aggregates, damaged organelles, intracellular pathogens, and lipid droplets in a process known as selective autophagy, which plays a critical role in the cellular response to disease [12]. In selective autophagy, specific cargo receptors recognize cellular components and mediate autophagosome formation [13].

VMP1 was first identified due to its high expression in acute pancreatitis [14]. We characterized VMP1 as an autophagy-related protein whose expression alone is sufficient to trigger autophagy [15]. VMP1 is essential for autophagosome formation [16,17]. Using the tissue-specific transgenic mouse model ElaI-VMP1, which constitutively expresses VMP1 in pancreatic acinar cells, we previously demonstrated that VMP1 expression induced by pancreatitis triggers a protective pathway that we termed zymophagy. This selective autophagic process recognizes, sequesters, and degrades prematurely activated zymogen granules within the pancreatic acinar cell, preventing further trypsinogen activation and cell death [18].

Considering that premature protease activation within acinar cells is critical in triggering local inflammatory responses in AP, we wondered whether VMP1-mediated selective autophagy could reduce the severity of the disease, particularly its harmful effects in other tissues such as the liver, lungs, and kidneys. To investigate this, we subjected ElaI-VMP1 mice to Hartwig’s model of SAP [7,8]. We examined the impact of zymophagy on pancreatic and extrapancreatic tissue damage in SAP, focusing on histopathology. Our results demonstrate that VMP1-mediated autophagy exerts a protective effect damping pancreatic and systemic histopathological damage that occurs in experimental severe acute pancreatitis.

## 2. Results

### 2.1. Intrapancreatic Features

#### 2.1.1. ElaI-VMP1 Mice Exhibit Lower Serum Enzyme Concentrations, Reduced Inflammatory Signs, and Less Necrosis During Experimental SAP

**Figure 1:**
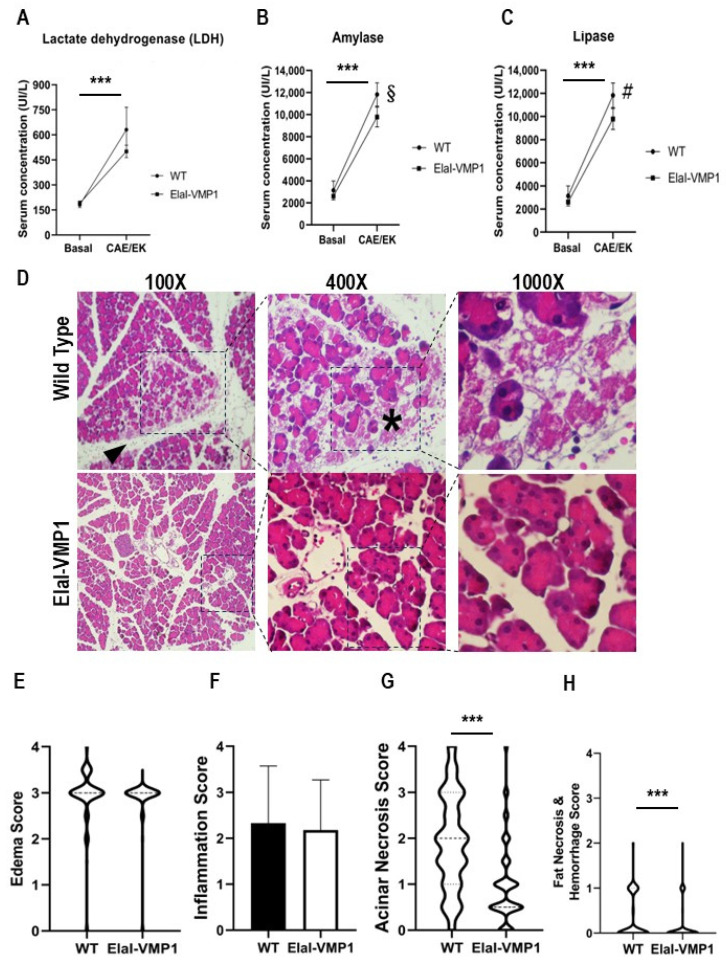
ElaI-VMP1 mice exhibit lower enzyme serum concentrations, reduced inflammatory signs, and less necrosis during experimental SAP. (**A**–**C**) Serum concentrations of LDH (**A**), amylase (**B**), and lipase (**C**) following CAE/EK administration. ***, *p* < 0.001 vs. untreated mice; §, *p* = 0.006 vs. wild-type (WT); #, *p* < 0.001 vs. WT. Bars represent mean ± SD, *n* = 4 per group. (**D**) Representative photomicrographs (100X, 400X or 1000X) of H&E-stained pancreatic tissue from ElaI-VMP1 and WT mice after CAE/EK administration, showing edema, inflammatory infiltration, and acinar necrosis. Inflammatory infiltration is present in the interstitial and inter-acinar spaces in WT mice (arrowhead) and around necrotic areas (asterisk). (**E**) Edema, (**F**) inflammation, (**G**) acinar necrosis, and (**H**) fat necrosis and hemorrhage scores in ElaI-VMP1 and WT mice after CAE/EK administration. ***, *p* < 0.001 vs. WT. For further details, refer to the text.

SAP is associated with varying degrees of pancreatic necrosis [8,19]. To assess pancreatic damage, we analyzed serum LDH concentration as a nonspecific marker of cellular necrosis before and after SAP induction in WT and ElaI-VMP1 mice. Both untreated groups showed similar LDH values before treatment, which increased following SAP induction (ElaI-VMP1: 190 ± 14 UI/L vs. 501 ± 37 UI/L; WT: 181 ± 16 UI/L vs. 631 ± 135 UI/L, *p* < 0.001). Although not statistically significant, Figure 1A indicates that the LDH increase was lower in ElaI-VMP1 mice.

A hallmark of AP is the elevation of amylase and lipase serum levels due to pancreatic acinar cell damage. Therefore, we analyzed the serum concentrations of these enzymes in WT and ElaI-VMP1 mice after pancreatitis induction. Untreated WT and ElaI-VMP1 mice had no significant differences in amylase (ElaI-VMP1: 2603 ± 180 UI/L vs. WT: 3130 ± 857 UI/L, *p* > 0.05) or lipase levels (ElaI-VMP1: 80 ± 11 UI/L vs. WT: 61 ± 8 UI/L, *p* > 0.05). After CAE/EK administration, both enzyme levels significantly increased compared to baseline (*p* < 0.001). However, the increase was significantly lower in ElaI-VMP1 mice compared to WT mice (amylase: 11,830 ± 1068 UI/L vs. 9780 ± 906 UI/L, *p* = 0.006; Figure 1B; lipase: 289 ± 57 UI/L vs. 160 ± 36 UI/L, *p* = 0.001; Figure 1C). These findings suggest that the pancreas in ElaI-VMP1 mice was less affected in the SAP model compared to WT mice.

To determine whether the lower enzyme levels correlated with reduced tissue injury, we performed a histopathological analysis. A total of 394 histological Hematoxylin and Eosin (H&E) photomicrographs from ElaI-VMP1 mice and 327 from WT mice were analyzed. CAE/EK administration caused structural alterations in the pancreas, confirming the tissue damage induced by SAP. Histologically, ElaI-VMP1 pancreata displayed significantly less damage than WT pancreata, which exhibited marked edema and hemorrhage (Figure 1D). In WT mice, areas of edema and necrosis were more prominent, along with inflammatory cell infiltration into acinar structures (arrowhead).

- Edema Analysis

The mean edema score for ElaI-VMP1 mice was 2.87 (SD: 0.53; SEM: 0.027; median: 3), whereas in WT mice, it was 2.93 (SD: 0.61; SEM: 0.034; median: 3; *p* = 0.1621). Although the difference was not statistically significant, Figure 1E shows that in ElaI-VMP1 mice, edema was primarily restricted to the interlobar and interlobular septa, whereas in WT mice, it extended into the interacinar and intercellular spaces (Figure 1D).

- Inflammatory Infiltration

Inflammatory cells were primarily located around blood vessels and necrotic areas (Figure 1D, arrowhead and asterisk). The mean inflammatory score for Elal-VMP1 mice was 2.18 (SD: 1.09; SEM: 0.055; median: 2, IQR: 1.5–3), whereas in WT mice, it was 2.33 (SD: 1.24; SEM: 0.069; median: 2, IQR: 1.5–3.5; *p* = 0.08 -Student’s *T* test; *p* = 0.047-non-parametric Mann–Whitney analysis). Although the mean inflammatory score did not differ significantly between ElaI-VMP1 and WT mice (Figure 1F), in ElaI-VMP1 mice inflammation was primarily focal (1 to 20 leukocytes/High Power Field-HPF-), whereas in WT mice diffuse infiltration (21 to 30 leukocytes/HPF) and microabscesses (>30 leukocytes/HPF) were observed (Figure 1D, 100Χ).

- Acinar Necrosis

Quantification of necrosis revealed that the mean acinar necrosis score was significantly lower in ElaI-VMP1 mice (mean: 0.99; SD: 0.88; SEM: 0.045; median: 0.5, IQR: 0.5–1.0) compared to WT mice (mean: 1.93; SD: 1.04; SEM: 0.057; median: 2.0, IQR: 1–3). As shown in Figure 1G, ElaI-VMP1 mice exhibited significantly fewer necrotic areas than WT mice (*p* < 0.001 in both parametric and non-parametric analyses). Additionally, WT pancreata displayed areas of extensive confluent necrosis (>16 necrotic cells/HPF).

- Fat Necrosis and Hemorrhage

Fat necrosis and hemorrhage were also evaluated. The mean fat necrosis score in ElaI-VMP1 mice was 0.076 (SD: 0.22; SEM: 0.011; median: 0, IQR: 0–0), while in WT mice, it was 0.18 (SD: 0.32; SEM: 0.018; median: 0, IQR: 0–0.5). The difference was statistically significant (*p* < 0.001) in both parametric and non-parametric analyses. Once again, ElaI-VMP1 mice exhibited significantly less necrosis than WT mice, as shown in Figure 1H.

#### 2.1.2. ElaI-VMP1 Mice Exhibit Higher Levels of Zymophagy, Which Significantly Correlates with Lower Acinar Necrosis During Experimental SAP

Microtubule-associated protein 1A/1B-light chain 3 (LC3) is a well-established autophagy marker as it remains attached to the autophagosome membrane, making it a reliable indicator of autophagy activity [11]. During pancreatitis, trypsin is the first zymogen to become activated [9]. In previous studies, we demonstrated that in ElaI-VMP1 mice, overstimulation of CCK-R with a supramaximal dose of CAE induces a selective form of autophagy known as zymophagy. This process selectively sequesters and degrades activated zymogen granules within the autolysosome in acinar cells, thereby preventing further trypsinogen activation and subsequent cell death [18].

**Figure 2:**
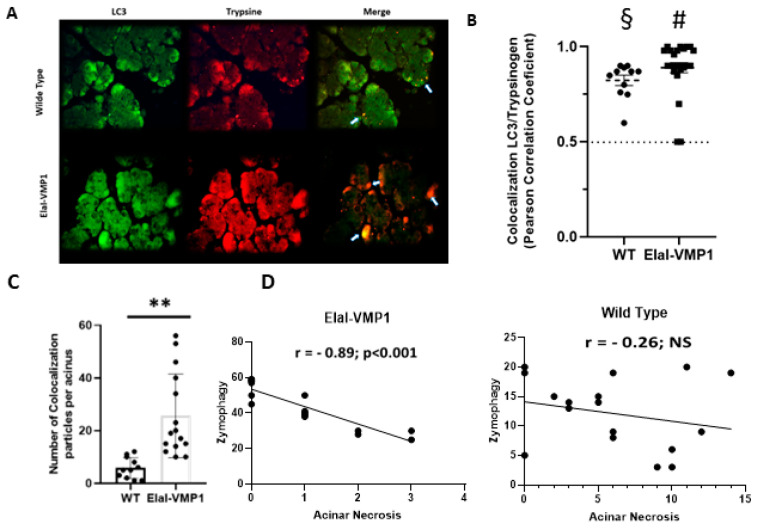
ElaI-VMP1 mice exhibit higher levels of zymophagy, which correlate significantly with lower levels of acinar necrosis following experimental SAP induction. (**A**) Representative microphotographs of immunofluorescence (IF) assays using anti-LC3 (green) and anti-trypsinogen (red) antibodies in ElaI-VMP1 and WT mice after CAE/EK administration. Some representative colocalization spots are marked with arrows. Magnification 400Χ. (**B**) Colocalization between LC3 and trypsinogen was quantified in at least 18 pancreatic acini per animal (*n* = 4). Each point on the graph represents Pearson’s correlation coefficient. § or #, *p* < 0.001, as determined by a two-tailed Student’s *t*-test compared to the theoretical mean of 0.5 (dash line). Error bars represent SEM. (**C**) Quantification of the number of colocalization spots, indicating the number of zymophagosomes per acinus, in at least 18 pancreatic acini of ElaI-VMP1 and WT mice. Error bars represent SD (*n* = 4). **, *p* < 0.01, as determined by a two-tailed Student’s *t*-test vs. WT mice. (**D**) Correlation between the number of zymophagosomes and the number of necrotic acinar cells per pancreatic acinus in ElaI-VMP1 and WT mice after SAP induction. Pearson’s correlation coefficient (r) and the level of significance (*p* < 0.001 and NS-Non Significant-respectively) are shown.

To analyze the potential relationship between zymophagy and histopathological features in severe experimental acute pancreatitis, we assessed the degree of colocalization between LC3 and trypsinogen following CAE/EK administration. Figure 2A shows colocalization events between LC3 and trypsinogen, indicative of the presence of zymophagosomes. To confirm these structures, we performed a colocalization test using ImageJ/Fiji and found that, in both mouse groups, the observed events corresponded to colocalization points between LC3 and trypsinogen (*p* < 0.001, two-tailed Student’s *t*-test vs. the theoretical mean of 0.5) (Figure 2B).

Next, we quantified the number of colocalization spots (i.e., the number of zymophagosomes) per acinus in ElaI-VMP1 and WT mice (Figure 2C). ElaI-VMP1 mice exhibited significantly more colocalization events between LC3 and trypsinogen compared to WT mice (*p* < 0.01, two-tailed Student’s *t*-test vs. WT), confirming that SAP triggers zymophagy, which is significantly enhanced in ElaI-VMP1 mice.

We then calculated the Pearson correlation coefficient between the number of zymophagosomes and the number of necrotic acinar cells per acinus in both mouse groups following SAP induction. In ElaI-VMP1 mice, there was a strong negative correlation (r = −0.83, *p* < 0.001), whereas in WT mice, the correlation was weak and not statistically significant (r = −0.26, *p* > 0.5) (Figure 2D).

Taken together, these results demonstrate that in this model of necrohemorrhagic pancreatitis, which causes extensive tissue damage in WT mice, ElaI-VMP1 mice develop only edematous pancreatitis. This reduced severity is significantly correlated with their enhanced ability to perform zymophagy.

### 2.2. Extrapancreatic Features

#### ElaI-VMP1 Mice Exhibited Preserved Liver Histology, Normal Kidney Histoarchitecture, and Clean Lung Tissue During Experimental SAP

The events that regulate the severity of acute pancreatitis remain largely unknown. There is a local inflammatory reaction at the site of damage, leading to a systemic inflammatory response syndrome. It is this systemic response, resulting in multi-organ failure, that is ultimately responsible for the morbidity and mortality of this disease [20]. To assess the severity of acute pancreatitis in WT and ElaI-VMP1 mice, we performed histopathological studies evaluating liver, kidney, and lung tissue damage.

- Liver

**Figure 3:**
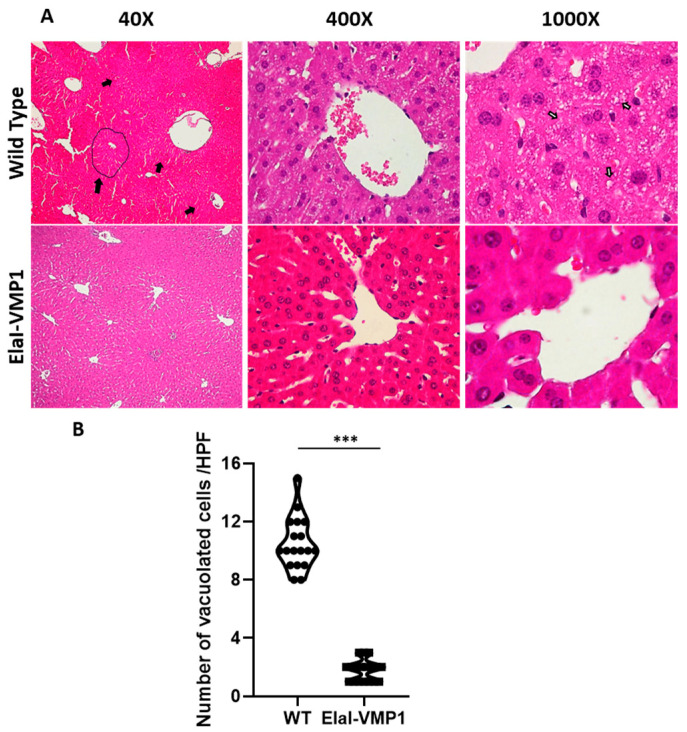
ElaI-VMP1 mice exhibit preserved liver histology during experimental SAP. (**A**) Representative images of liver tissue from WT and ElaI-VMP1 mice after CAE/EK administration, stained with H&E. Images at LPF (40Χ) and HPF (400Χ and 1000Χ) show representative necrotic areas (black arrows and marked area at LPF) and cells with intracytoplasmic vacuoles (white arrows at HPF 1000Χ). (**B**) Quantification of vacuolated hepatic cells in at least 18 HPF of both ElaI-VMP1 and WT mice after SAP induction. ***, *p* < 0.001 vs. WT.

Morphological evaluation of the liver after CAE/EK-induced SAP revealed that ElaI-VMP1 mice maintained normal histoarchitecture, unlike WT mice, which exhibited numerous areas of necrosis, particularly around the central vein, accompanied by neutrophilic infiltration (Figure 3A, Low Power Field-LPF-, left). Focusing on the necrotic areas at HPF, we observed that in ElaI-VMP1 mice, hepatocytes were preserved, whereas, in WT mice, cells showed evident signs of necrosis, such as swollen cytoplasm and numerous intracytoplasmic vacuoles (Figure 3A, HPF, center and right). Figure 3B shows the quantification of vacuolated cells, where ElaI-VMP1 mice displayed a significantly lower number of vacuolated cells compared to WT mice (mean ± SD, ElaI-VMP1: 1.78 ± 0.73 vs. WT: 10.5 ± 1.79; *p* < 0.001), confirming the preservation of liver histology.

- Kidneys

**Figure 4:**
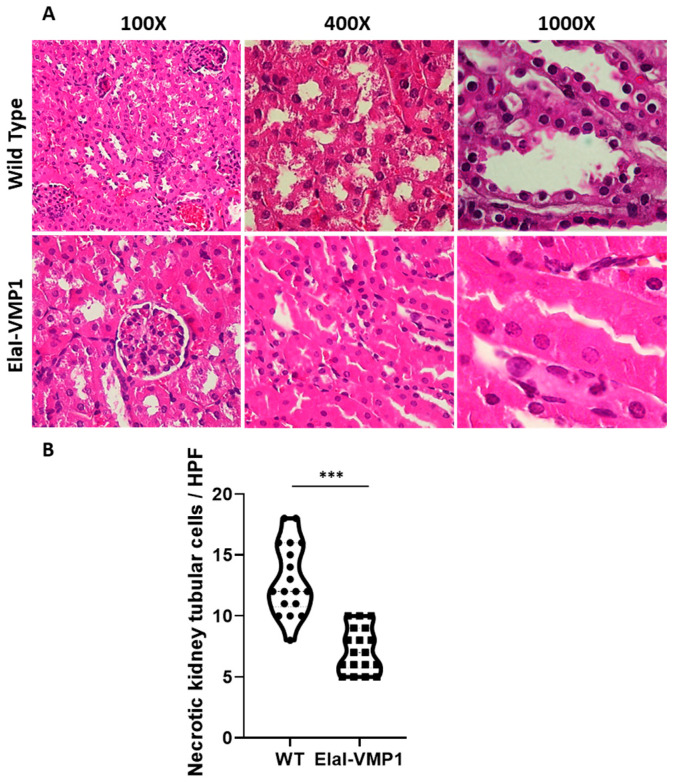
ElaI-VMP1 Mice Exhibit Normal Kidney Histoarchitecture Without Mesangial Proliferation After Experimental SAP. (**A**) Representative images of kidney tissue from WT and ElaI-VMP1 mice after CAE/EK administration, stained with H&E. Magnification 100Χ, 400Χ or 1000Χ. (**B**) Quantification of necrotic tubular cells in at least 18 HPF of both ElaI-VMP1 and WT mice after SAP induction. ***, *p* < 0.001 vs. WT.

Regarding kidney evaluation, after CAE/EK administration, the histoarchitecture of the kidney was preserved in ElaI-VMP1 mice, although isolated areas of tubular necrosis with debris in the lumen were observed. In contrast, SAP induction in WT mice resulted in large areas of acute tubular necrosis with patchy loss of tubular epithelial cells, desquamation, pyknosis, and loss of nuclei. Additionally, we observed the presence of intraluminal cellular debris (Figure 4A). Quantifying necrotic tubular cells at HPF (400Χ), we found that ElaI-VMP1 mice had significantly fewer necrotic cells compared to WT mice (mean ± SD, ElaI-VMP1: 7.22 ± 1.83 vs. WT: 13.00 ± 2.93; *p* < 0.001) (Figure 4B). Furthermore, glomeruli from WT mice exhibited mesangial proliferation, a condition not observed in ElaI-VMP1 mice.

- Lungs

**Figure 5:**
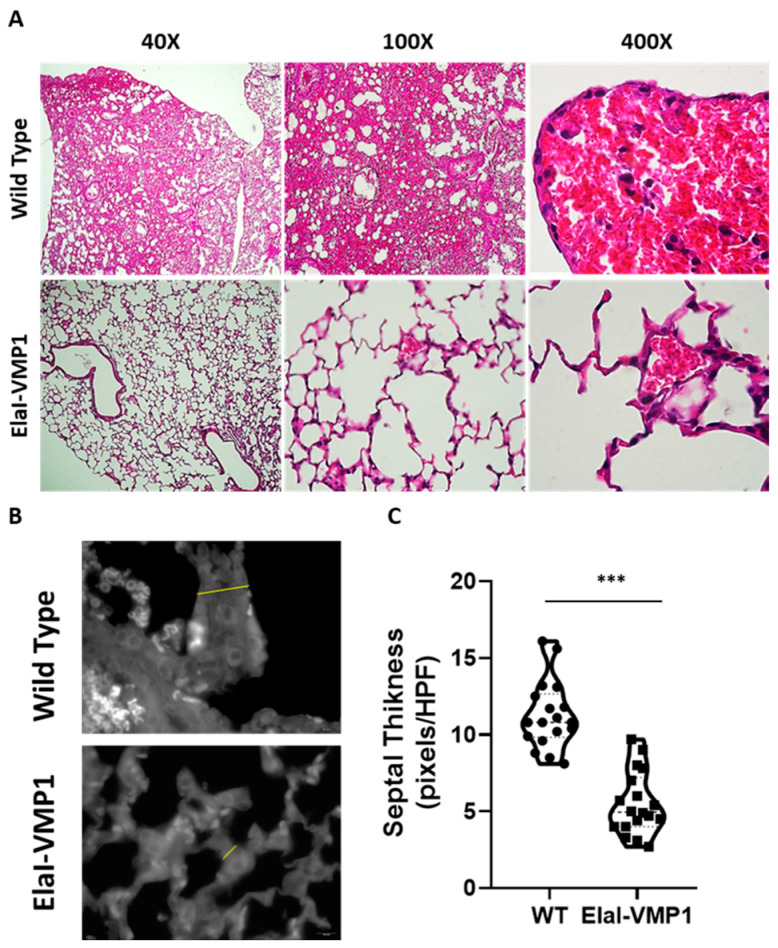
ElaI-VMP1 mice show clean lung tissue and reduced septal thickness during experimental SAP. (**A**) Representative images of lung tissue from WT and ElaI-VMP1 mice after SAP induction, stained with H&E. Magnification 40Χ, 100Χ or 400Χ. (**B**) Representative images of lung tissue from WT and ElaI-VMP1 mice after SAP induction, stained with Negative stain (magnification 1000Χ). Thickness of alveolar–capillary septum is marked with yellow lines in panel (**B**). (**C**) Quantification of septal thickness from at least 18 HPF per animal after CAE/EK administration in ElaI-VMP1 and WT mice. ***, *p* < 0.001 vs. WT.

Finally, the analysis of lung involvement in this experimental model of SAP is shown in Figure 5. ElaI-VMP1 mice presented mostly clear lung tissue, whereas WT mice exhibited numerous hemorrhagic foci within the alveolar spaces, associated with numerous infiltrating neutrophils (Figure 5A). Regarding alveolar capillary septum thickness as an indicator of inflammation, we found that CAE/EK administration caused significantly less widening in ElaI-VMP1 mice than in WT mice (mean ± SD, ElaI-VMP1: 56.56 ± 19.45 vs. WT: 112.78 ± 21.99 pixels/HPF; *p* < 0.01, Student’s *t*-test), as shown in Figure 5B,C.

In summary, the results obtained from the ElaI-VMP1 mice indicate that zymophagy functions as a protective cellular mechanism, preventing the severity of AP.

## 3. Discussion

ElaI-VMP1 mice are transgenic mice in which the acinar-cell-specific elastase promoter drives VMP1 expression and autophagy, without any alteration in pancreatic tissue [15]. The induction of AP by hyperstimulation of CCK-R with CAE triggers zymophagy in these mice, preventing the spread of zymogen activation and cell death [18]. In this study, we used Hartwig et al.’s model [8] of necrohemorrhagic AP, induced by simultaneous infusion of CAE and EK, in WT and ElaI-VMP1 mice. This model is characterized by severe local and systemic organ injury, with marked intrapancreatic protease activation. Our results show that, although the necrohemorrhagic model of AP is characterized by severe local and systemic organ injury, in ElaI-VMP1 mice, the severity of the disease was significantly reduced. These findings suggest that zymophagy, the selective autophagy mediated by VMP1 expression, functions as a protective cellular mechanism, preventing the severity of AP.

As previously reported in ElaI-VMP1 mice subjected to CAE-induced pancreatitis [18], we found that in the SAP model, ElaI-VMP1 mice exhibited dramatically less pancreatic tissue damage (i.e., acinar or fat necrosis, hemorrhagic foci, and inflammatory infiltration) (Figure 1). Furthermore, we evaluated extrapancreatic features characteristic of SAP in the lungs, liver, and kidneys, as markers of systemic injury and disease severity.

Histopathological analysis of the liver and kidneys revealed a similar pattern, with a clear reduction in tissue damage in ElaI-VMP1 mice. Focusing on liver tissue, as part of the pancreatic blood flow, it has been reported that high concentrations of activating enzymes and inflammatory mediators reach the liver early during AP, and pancreatic elastases can switch on Kupffer cells to produce cytokines by activating the nuclear transcription factor-κB (NF-κB) pathway during SAP [21]. Additionally, pancreatitis causes disruption of the intestinal barrier, allowing endotoxins to enter the bloodstream and invade the liver, where they activate phospholipase A2, leading to membrane phospholipid degradation and inducing free radicals that mediate lipid peroxidation in liver cells [22]. In our study, we found that while the liver histoarchitecture and hepatocytes were preserved in ElaI-VMP1 mice, hepatocytes of WT mice exhibited swollen cytoplasm and numerous intracytoplasmic vacuoles, indicative of cellular necrosis (Figure 3).

Regarding kidneys, acute kidney injury is one of the main complications of SAP [23]. However, we found that ElaI-VMP1 mice maintained conserved kidney histoarchitecture, with only isolated areas of tubular necrosis and debris in the lumen, in contrast to WT mice, which exhibited large areas of acute tubular necrosis, desquamation, pyknosis, and loss of nuclei—typical damage seen in acute renal failure associated with SAP (Figure 4). It has been proposed that kidney injury following SAP is associated with a complex process, which involves the release of cytokines and damage-associated molecular patterns from dying acinar cells into the bloodstream. However, the precise underlying mechanisms remain to be elucidated [24].

Acute respiratory distress syndrome is a systemic inflammatory complication commonly associated with pancreatitis, which is triggered by neutrophil invasion of the lungs [7]. Normal lungs exhibit thin septa with few leukocytes. Our results showed that ElaI-VMP1 mice had clear lung tissue with thinner septa than WT mice. Furthermore, WT mice had several hemorrhagic foci in the alveolar spaces with numerous infiltrating neutrophils (Figure 5).

Several reported studies have suggested that the acinar cell response to injury may, itself, be an important determinant of disease severity. In these studies, mild AP was found to be associated with extensive apoptotic acinar cell death, whereas SAP was found to involve extensive acinar cell necrosis but very little acinar cell apoptosis. [25]. Trypsinogen activation is widely accepted as one of the early events in the pathophysiology of AP [9], leading to necrosis and the interstitial spreading of activated zymogen, transforming mild edematous pancreatitis into a severe necrohemorrhagic disease [26,27]. Our results show that the selective autophagic pathway, zymophagy, is highly activated in pancreatic acinar cells from ElaI-VMP1 mice during the SAP model and significantly correlates with the rate of necrosis in pancreatic tissue. The reduction in pancreatic tissue damage is translated into a lower level of systemic injury in ElaI-VMP1 mice evidenced by less damage to liver, kidney, and lung tissue. Moreover, the protective cellular response mediated by VMP1 expression would explain, at least in part, the self-limiting nature of the disease in most cases. In contrast, the severe forms of pancreatitis may present an excess load and accelerated degradation demand that could overwhelm or alter the protective capacity of this selective autophagic process.

Zymophagy was also characterized as a defense mechanism of the acinar cell in human pancreatitis. Using IF assays for VMP1, LC3, and trypsinogen, Grasso et al. demonstrated the formation of autophagosomes where LC3 and zymogen granules colocalize in samples of patients with AP. Furthermore, autolysosomal structures at different levels of maturation were found in the progression of autophagy in the patient’s samples by IF of Lamp2, a specific lysosomal marker. This demonstrates that zymophagy ultimately leads to the complete degradation of zymogen granules sequestered within the autolysosome. These findings in human samples collectively support that zymophagy is an evolutionarily conserved mechanism of cellular response to disease [18]. Recently, Zheng et al. (2022) [28] found that FXR (Farnesoid X receptor), a ligand-activated transcription factor that plays a crucial role in regulating glucose, lipid, bile acid, and amino acid metabolism in the liver and intestine, also has an anti-inflammatory effect in various inflammatory diseases. In pancreatic tissue from human patients with pancreatitis, they observed a marked increase in nuclear FXR. In this study, Osgin1 (Oxidative stress-induced growth inhibitor 1) was identified as a direct target of FXR in the exocrine pancreas, and its expression was also increased in human pancreatitis tissues compared with normal pancreatic tissues. Finally, they demonstrated that the FXR-Osgin1 axis stimulated autophagic flux in pancreatic tissues and cell lines. Furthermore, they found that suppression of FXR or inhibition of pancreatic Osgin1 led to more severe pancreatitis in murine models of CAE-induced AP. Our results are consistent with these findings, highlighting the importance that preserved and active autophagy mitigates progression to severe pancreatitis.

Several pharmacological inducers of autophagy have been developed and studied in the field of pancreatic cancer [29] but much less is known regarding AP. For example, in a model of traumatic pancreatitis, the effect of the biological agent ucMSC-Ex (umbilical cord mesenchymal stem cell exosomes) attenuates pancreatic damage, activating autophagy in pancreatic acinar cells by inhibiting mTOR [30]. In another study, autophagic flux is impaired in hypertriglyceridemia (HTG)-induced AP models and appears to be closely linked to endoplasmic reticulum stress. Rapamycin may prevent the worsening of HTG-associated acute pancreatitis by inhibiting the mTORC1/S6K1 pathway [31]. However, a definitive conclusion on the effects of inducers remains to be achieved. A major challenge in elucidating these effects is that autophagy-modulating drugs, such as rapamycin, act on a wide range of molecules or pathways and cannot be considered selective for the autophagy pathway alone.

Consequently, the search for more specific agents to modulate autophagy, and particularly zymophagy, is a promising direction in the management of AP to prevent the severity of this disease. Recently, we identified ubiquitination as a post-translational modification of VMP1 from the initial steps of autophagosome biogenesis from in vitro studies with human tumor cells. VMP1 remains ubiquitinated as part of the autophagosomal membrane throughout the autophagic flux until autolysosome formation. VMP1 ubiquitination is decreased by the cullin-RING ubiquitin ligase inhibitor MLN4924 and increased by overexpression of the cyclic protein cdt2, modulating VMP1 recruitment and autophagosome formation [32]. Therefore, it would be interesting to explore the modulation of ubiquitin ligase as a regulator of VMP1-mediated autophagy in the context of AP in further research.

Another interesting objective is to predict whether AP will progress to severity or not. The severity of the disease is associated with the degree of necrosis and this condition with the induction of proinflammatory cytokines, which are thought to be responsible for systemic injury [2]. The reduced systemic impact in ElaI-VMP1 mice seems to be entirely attributed to the lower pancreatic damage observed in these animals. However, Carrascal et al. (2022) demonstrated that the release of molecules in vesicles in the early stages of AP predicts the severity of the disease [33]. In this regard, using a cellular model of AP, we recently discovered that VMP1 is secreted in vesicles in response to pancreatic acinar cell injury [34]. Thus, these findings suggest that VMP1 could be evaluated as a biological marker related to the severity of the disease.

In summary, we describe for the first time that in ElaI-VMP1 mice, the severity of the disease is completely attenuated and correlates with the capacity to develop effective Ezymophagy mediated by VMP1. These results confirm that zymophagy functions as a protective pathophysiological mechanism against both pancreatic and extrapancreatic tissue injury. Further studies on the mechanism of VMP1-mediated selective autophagy in the context of AP are necessary to determine its relevance and possible modulation in the prevention of the severity of this disease in humans.

## 4. Materials and Methods

- Mice

Male C57BL6J mice (referred to as WT) and C57BL6J-ElaI-VMP1 transgenic mice (referred to as ElaI-VMP1) weighing 20–25 g were used in the experiments. The animals were housed with free access to food and water. Animal experiments were approved by the Animal Care and Research Committee of the School of Pharmacy and Biochemistry, University of Buenos Aires, and strictly followed the International Guiding Principles for Biomedical Research Involving Animals.

- Transgenic Mice (ElaI-VMP1 Mice)

Transgenic mice were kindly provided by Dr. Juan Iovanna (INSERM U1068, Marseille, France). The transgene cassette was constructed using the pBEG vector (Strategene, La Jolla, CA, USA) [35]. Briefly, the expression cassette contains the acinar-specific control region (−500 to +8) from the rat elastase I gene and the human growth hormone 3′-untranslated region (+500 to +2657). This construct was digested with BamHI (Madison, WI, USA), filled in, dephosphorylated, and ligated with rat VMP1-EGFP released from the pEGFPVMP1 plasmid. A 1940 kb HindIII/NotI fragment was isolated and used for microinjections into inbred FVB zygotes. Genomic DNA was prepared and tested by Southern blot and PCR. VMP1 expression was confirmed by immunohistochemistry and immunoblot in pancreatic acinar cells of transgenic mice [15].

- CAE/EK-induced Pancreatitis Model

WT or ElaI-VMP1 male mice were fasted for 12–16 h with free access to water. SAP was induced by 7 hourly intraperitoneal injections of 50 μg/kg CAE. Starting from the second injection of CAE, 30 IU/kg of EK was also administered; i.e., animals received 7 injections of CAE and 5 injections of EK. Mice were euthanized 9 h after the first injection. Both CAE and EK were purchased from Sigma-Aldrich (St Louis, MO, USA).

- Biochemical Markers

Animal serum amylase, lipase, and LDH activity were measured by Cobas^®^ c 501 Analyzer (Roche, Indianapolis, IN, USA) and are expressed as units per liter.

- Histological Studies
  ○ Pancreatic Tissue

For histological analysis, the pancreas was removed, and tissues were fixed in 4% paraformaldehyde in phosphate-buffered saline (PBS) pH 7.4, embedded in paraffin, and sectioned at 5 μm thickness. One set of tissues was stained with H&E, while another set was prepared for IF assays. The severity of pancreatitis was evaluated in a blind manner using at least 18 HPF (i.e., 400Χ) per animal. To score the intensity of damage in pancreatic tissues we perform a morphometric analysis based on Schmidt’s histopathologic criteria [36]. The scoring system (0 to 4) is presented in Table 1.

 ○ Extra-pancreatic Tissue Damage

To assess the systemic impact caused by SAP, we evaluated histological changes and the degree of damage to liver, kidney, and lung tissue.

1. Liver Damage: At LPF (i.e., 40×), the presence and localization of necrotic areas (e.g., centrilobular) were considered. At HPF, histological evidence of loss of hepatocyte membrane integrity, signs of tissue necrosis (e.g., cytoplasm swelling and/or intracellular vacuoles), and areas of leukocyte infiltration, especially in centrilobular areas, were examined;
2. Kidney Damage: Kidney damage was assessed by examining areas of acute tubular necrosis with patchy loss of tubular epithelial cells, desquamation, pyknosis, and loss of nuclei, as well as the presence of intraluminal cellular debris;
3. Lung Damage: For lung damage evaluation, particular emphasis was placed on septal integrity and thickness, as well as hemorrhagic foci in the alveolar spaces. The presence of leukocyte infiltration was noted, though it was not the central focus of the lung damage assessment.

- Antibodies

Polyclonal sheep anti-LC3 (Abcam, Cambridge, UK) antibody was used according to the manufacturer’s instructions. Polyclonal goat anti-trypsin (Santa Cruz Biotechnology, Santa Cruz, CA, USA) antibody was used according to the manufacturer’s instructions. Alexa Fluor 488 and 594 antibodies (Molecular Probes, Waltham, MA, USA) were used for IF assays.

- IF Assays

Tissue sections (5 μm) were washed several times with PBS. Samples were permeabilized with triton X-100 (Sigma-Aldrich, St Louis, MO, USA) 0.1% in PBS for 5 min, washed three times with PBS, and blocked with fetal bovine serum 10% in PBS for 1 h. Then, tissue sections were incubated with primary antibodies overnight at 4° C. The next day, the samples were incubated with secondary antibodies for 2 h at room temperature. Then, the samples were mounted in 1,4-diazabicyclo [2.2.2] octane (Sigma-Aldrich, St Louis, MO, USA) and observed using an LSM Olympus FV1000 microscope with a UPLSAPO 60X objective (NA: 1.35) (Olympus America Inc., Center Valley, PA, USA).

- Statistics

Changes in enzyme concentrations before and after the experimental intervention were evaluated by two-way ANOVA, with repeated measures in one of the factors (Bonferroni Post Hoc Test). Differences between two groups of independent quantitative data were assessed using Student’s *t*-test and/or the non-parametric Mann–Whitney test, depending on the data distribution. In some cases, both tests were applied. In cases where both tests were significant (*p* < 0.05), only the significance of the parametric test is reported. When significance differs between the parametric and non-parametric tests, the significance of each test is reported separately. The Pearson correlation coefficient was calculated to assess whether zymophagy was correlated with the degree of acinar necrosis. Statistical significance is indicated as follows: NS, non-significant; *, *p* <0.05; **, *p* < 0.01; ***, *p* < 0.001.

## Figures and Tables

**Table 1:** Histopathological scoring criteria.

Histopathological Score	0	1	2	3	4
Edema	Absent	Diffuse expansion of Interlobar septae	“1” + Diffuse expansion of Interlobular septae	“2” + Diffuse expansion of Interacinar septae	“3” + Diffuse expansion of intercellular spaces
Inflammation and perivascular infiltrate	0–5 intralobular or perivascular leukocytes/HPF	6–15 intralobular or perivascular leukocytes/HPF	16–25 intralobular or perivascular leukocytes/HPF	26–35 intralobular or perivascular leukocytes/HPF	>35 intralobular or perivascular leukocytes/HPF
Acinar necrosis	Absent or Focal occurrence of 0–4 necrotic cells/HPF	Diffuse occurrence of 0–4 necrotic cells/HPF or Focal occurrence of 5–10 necrotic cells/HPF	Diffuse occurrence of 5–10 necrotic cells/HPF or Focal occurrence of 11–16 necrotic cells/HPF	Diffuse occurrence of 11–16 necrotic cells/HPF or Focal occurrence of >16 necrotic cells/HPF	>16 necrotic cells/HPF
Hemorrhage and fat necrosis	Absent	1–3 foci	4–5 foci	6–7 foci	≥8 foci

## Data Availability

The original contributions presented in this study are included in the article. Further inquiries can be directed to the corresponding author(s).

## Abbreviations

The following abbreviations are used in this manuscript:

ANOVA	Analysis of Variance
AP	Acute Pancreatitis
CAE	Caerulein
CCK	Cholecystokinin
CCK-R	Cholecystokinin Receptor
EK	Enterokinase
ElaI-VMP1	C57BL6J-ElaI-VMP1 transgenic mice
FXR	Farnesoid X receptor
H&E	Hematoxylin and Eosin
HPF	High-Power Field
IF	Immunofluorescence
IQR	Interquartile Range
Lamp2	Lysosome-Associated Membrane Glycoprotein 2
LC3	Microtubule-Associated Protein 1A/1B-light chain 3
LDH	Lactate Dehydrogenase
LPF	Low-Power Field
mTOR	Mammalian Target of Rapamycin
mTORC1	Mammalian Target of Rapamycin Complex 1
NF-κB	Nuclear Transcription Factor-κB
NS	Non-Significant
Osgin1	Oxidative Stress-Induced Growth Inhibitor 1
PBS	Phosphate-Buffered Saline
S6K1	Serine/threonine Kinase 1
SAP	Severe Acute Pancreatitis
SD	Standard Deviation
SEM	Standard Error of the Mean
VMP1	Vacuole Membrane Protein 1
WT	Wild-Type
